# The impact of medical student research as a discussion topic during the residency interview process

**DOI:** 10.1186/s12909-021-02989-x

**Published:** 2021-11-01

**Authors:** Kelly Daus, Matthew McEchron

**Affiliations:** University of Arizona College of Medicine - Phoenix, 475 N 5th St, Phoenix, AZ 85004 USA

**Keywords:** Medical education, Scholarly research, Residency interview, Residency match

## Abstract

**Background:**

Students with a greater number of research experiences are more successful in the National Residency Match Program (NRMP.) As a result, approximately two-thirds of allopathic medical schools have implemented a scholarly research project (SP) as a part of their curriculum. While inclusion of an SP in the medical school curriculum increases research productivity, literature to date has not investigated the frequency with which it is a discussion topic during residency interviews.

**Methods:**

One hundred twenty-three students from the graduating class of 2019 and 2020 at the University of Arizona College of Medicine - Phoenix (UACOMP) completed a 17-question survey examining the student’s SP and whether they completed additional research, with an overall response rate of 82.6%. Survey participants were asked to quantify how many residency interviewers asked about their SP or additional research during the interview process.

**Results:**

Twenty-seven percent of interviewers asked students about their SP and 41% of interviewers asked students about additional non-SP research. 40% of interviewers asked about research overall to include SP and/or non-SP research. A greater percentage of interviewers (50%) asked students about their SP if they had undertaken additional research compared to interviewers of students who did not undertake additional research (29%, *p* = 0.0237). A greater percentage of interviewers at academic institutions (31%) asked students about their SP, compared with a smaller percentage of interviewers at predominantly non-academic programs (22%, *p* = 0.0054). There were no significant differences in the proportion of interviewers asking about the SP based on the type of specialty, competitiveness of specialty, relatedness project topic to specialty, and publication/presentation status of project.

**Conclusion:**

Student research experiences may serve as a frequent discussion topic during the residency interview. Approximately one-quarter of interviewers ask about the SP regardless of specialty, research topic, and publication/presentation status of the project. Students with additional research experiences beyond their SP may experience a higher percentage of interviewers asking about their SP. Also, students applying to predominantly academic programs may experience a higher proportion of interview questions about research compared to peers interviewing at non-academic programs.

## Background

Throughout the current body of medical education literature various models of incorporating scholarly research within the undergraduate medical education curriculum have been described as well as their impact on student success [1–6]. Previous research has shown that osteopathic and allopathic medical students matching into residency programs have a significantly greater number of research accomplishments than unmatched applicants [7]. Although causation cannot be concluded from several studies in this area, there is clearly an association between research experience and increased match success [8, 9]. In the 2020 Match, U.S. M.D. seniors reported an average of 3.6 research experiences [10]. For almost all specialties, matched U.S. MD seniors had on average greater numbers of research experiences, research conference presentations, and publications compared to students who did not successfully match [10]. For specialties including dermatology, interventional radiology, neurosurgery, otolaryngology, orthopedic surgery, and radiation oncology, where the large number of applicants relative to the number of positions available is more pronounced, the number of research experiences undertaken is even greater [10]. Even prior to the match, authorship of one or more publications is associated with a greater number of interview invitations for integrated plastic surgery applicants [11] and over 90% of general surgery program directors state that they consider basic and clinical research almost always or all the time when evaluating candidates [12].

As medical students with a higher number of research experiences are more successful in the Match, almost two-thirds of allopathic medical schools have implemented a scholarly research project as a component of their curriculum with approximately one-third of schools making completion of this scholarly research project mandatory for graduation [13]. The University of Pittsburg School of Medicine assessed the research productivity of its students before and after the implementation of a mandatory scholarly project (SP) and while noting only a modest rise in the number of students engaged in research, they identified a significant increase in the number of students with publications and first authorship [14]. Research experience, publications, and conference presentations clearly impact an applicant’s success in the Match, and this may motivate medical schools to make more research opportunities available for students.

Some objectives of mandatory scholarly research projects are to produce critical thinking life-long learners with self-directed independent learning skills, writing skills, and an understanding of the scientific method [13]. These key objectives of SP curricula are also highly desired characteristics in residency applicants [15]. The NRMP data suggest that research experience is an important component of the residency application [10]. This study sought to determine the frequency at which interviewers asked applicants about their SP during the residency interview process. We hypothesized that regardless of the research topic or publication outcome, a student’s scholarly research project would be a frequent discussion topic during the interview.

## Methods

The University of Arizona College of Medicine-Phoenix (UACOMP) requires students to complete a mandatory 4-year, longitudinal SP that is hypothesis driven and culminates in a poster presentation and written thesis. The present study surveyed fourth year medical students from the graduating class of 2019 and 2020 at UACOMP using an online survey approximately 2 months following completion of residency interviews and 2 weeks prior to the Match. Students were allotted 20 min during an in-person session on campus to complete the 17-question survey. This survey study was approved by the Institutional Review Board at the University of Arizona. Survey completion was optional, anonymous, and all participants agreed electronically to an informed consent prior to beginning the survey and could exit the survey at any time.

**Fig. 1:**
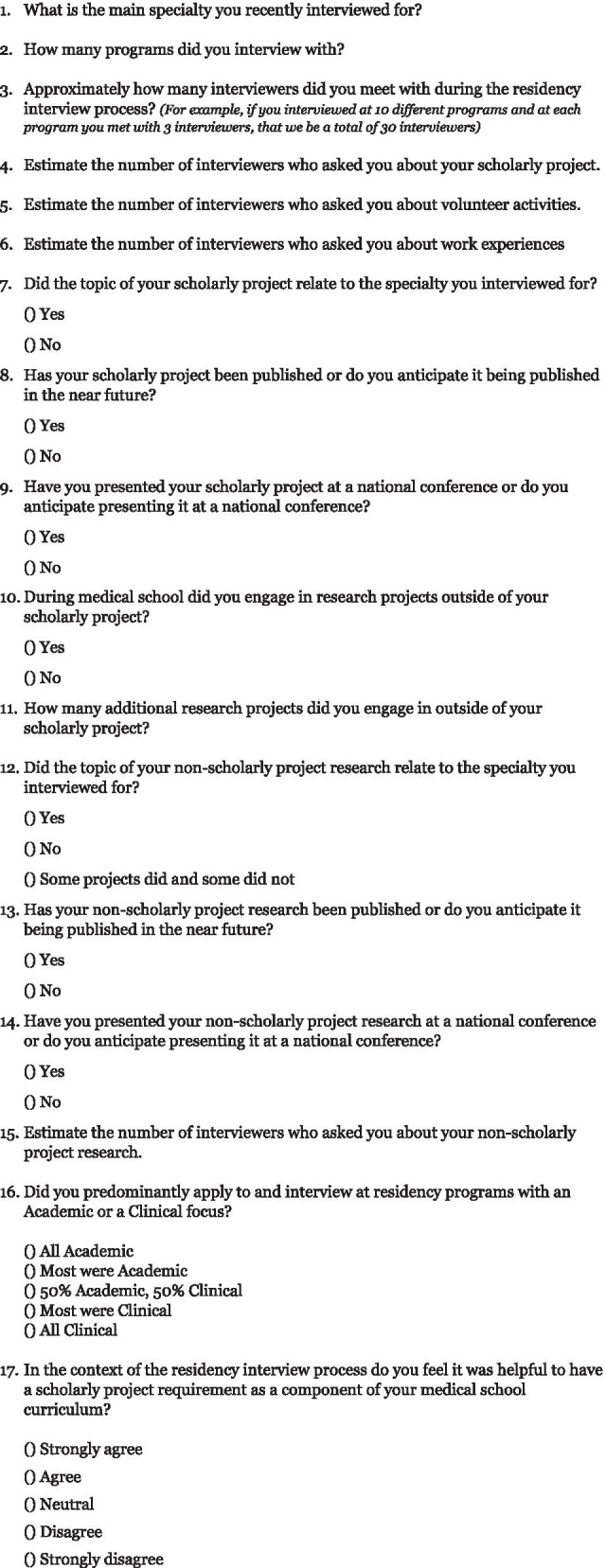
Study Survey

The survey examined the student’s SP, volunteer experiences, work experiences, and non-SP research. The survey also determined if students published or presented research at a national conference, and whether the research topics related to the specialty sought by the medical student. Students were asked to estimate the number of interviewers they met with during the interview process and the number of interviewers who asked them about their SP, volunteer experiences, work experiences, and non-SP research. The survey also gathered baseline characteristics of students including specialty and predominant type of program the student interviewed with – academic or community. A full list of survey questions can be found in Fig. 1.

### Statistical analysis

Survey results were collapsed across both classes to form a total survey population of 123 participants as there were no significant differences between the classes. Publication, presentation at a national conference, and topic relatedness of research to chosen specialty for both SP and non-SP research were reported as frequencies with percentages. The proportion of interviewers asking about SP, volunteer experiences, work experiences and non-SP research was calculated for each survey participant. These were then treated as a continuous variable and reported as means with standard deviations. Two-proportion z-tests with a significance level of 0.05 was used to determine whether the hypothesized difference between proportions of interviewers asking about SP differed significantly based on specialty type, competitiveness of specialty, publication of SP, presentation of SP, topic of SP, undertaking of additional non-SP research, and application to predominantly academic programs.

## Results

### Baseline characteristics

A total of 123 students participated in the survey from the graduating class of 2019 and 2020 at the UACOMP. A high percentage of the students completed the survey, with 62 out of the 67 students in the class of 2019 (92.5%) and 61 out of the 82 students in the class of 2020 (74.3%). The overall survey response rate was 82.6%. The baseline characteristics for the survey participants from the Class of 2019 and 2020 can be found in Table 1. There were no significant differences in baseline characteristics with roughly two-fifths of the survey population interviewing for primary care (40.7%) and three-fifths in non-primary care specialties (59.3%). Approximately one-fifth of the survey participants (18.7%) interviewed in more competitive specialties designated by having an annual number of applicants per position greater than 1.35 [10].

**Table 1:** Baseline Characteristics

	Class of 2019	Class of 2020	Total
**Number of Students**	62	61	123
**Primary Care**	30	20	50 (41%)
Family Medicine	7	7	14
Internal Medicine	14	6	20
Internal Medicine-Pediatrics	1	0	1
Pediatrics	8	7	15
**Non-Primary Care**	32	41	73 (59%)
Anesthesiology	7	2	9
Child Neurology	0	1	1
Diagnostic Radiology	5	4	9
Emergency Medicine	7	12	19
General Surgery^a^	1	3	4
Neurology	1	1	2
Neurosurgery^a^	2	0	2
OB/GYN	3	2	5
Ophthalmology^a^	0	2	2
Orthopedic Surgery^a^	1	5	6
Otolaryngology^a^	2	3	5
Pathology	1	0	1
Plastic Surgery^a^	0	2	2
Psychiatry	1	3	4
Urology^a^	1	1	2

^a^ more competitive specialties denoted by annual number of applicants per position > 1.35

All students completed the mandatory SP curriculum, and their project title was included in their Medical Student Performance Evaluation (MSPE) letter as part of their residency application. The research characteristics of students’ SP and non-SP research including publication status, presentation at a national conference, and topic relatedness to chosen specialty can be found in Table 2. Almost half of students reported publishing their SP (48%), and one-third presented their SP at a national conference. The survey showed that 65% of students reported undertaking additional research not related to their mandatory SP. Over three-fourths of these students published their additional research in peer-reviewed journals (78%) and approximately half (48%) presented this additional research at a national conference.

**Table 2:** Research Characteristics

	Total Number of Students (%)
**Scholarly Project**	123 (100%)
Published	59 (48%)
Presented at National Conference	41 (33%)
Topic Related to Chosen Specialty	58 (47%)
**Additional Research**	80 (65%)
Published	62 (78%)
Presented at National Conference	39 (49%)
Topic Related to Chosen Specialty	62 (78%)

### SP impact on the interview conversation

The survey revealed that on average 40% of interviewers (SD 30.7) asked students about their research experiences, while 41% of interviewers (SD 42.7) asked students about volunteering experiences, and 35% of interviewers (SD 32.9) asked students about work experiences as shown in Table 3. Specifically, with regard to the type of research discussed, an average of 27% (SD 27.0) and 41% (SD 32.0) of interviewers asked about the student’s SP and additional research, respectively.

**Table 3:** Mean Reported Proportion of Interviewers Asking about Research, Volunteering & Work Experience

	Proportion of Interviewers (%, SD)
**Research Overall**	40% (30.7)
Scholarly Project	27% (27.0)
Additional Research	40% (32.0)
**Volunteering**	41% (42.7)
**Work Experiences**	35% (32.9)

Survey data was analyzed to determine if the proportion of interviewers asking about a student’s SP differed based on seven factors shown in Table 4. There were no significant differences between the proportion of interviewers asking about a student’s SP based on the type of specialty or competitiveness of the specialty. Additionally, there were no significant differences between the proportion of interviewers asking about a student’s SP based on the publication or presentation status of the project or whether the topic was related to the student’s chosen specialty. However, the amount of research experience undertaken by the student outside of the SP had a significant impact on the proportion of interviewers asking about the SP. Results showed that 50% of interviewers (SD 26.2) asked students about their SP if they had undertaken additional research outside of their SP compared to 29% of interviewers (SD 28.4) of students who did not undertake additional research (*p* = 0.0237). Furthermore, the predominant type of programs the student interviewed with (i.e., programs with an academic versus community focus) had a significant impact on the proportion of interviewers who asked about the SP. Students applying to “almost all” or “more than half” of programs with an academic or research focus had a greater proportion of interviewers (31%, SD 27.9) asking them about their SP versus interviewers of students (22%, SD 25.5) who reported applying to “none at all” or “less than half” of programs with an academic research focus (*p* = 0.0054).

**Table 4:** Factors Impacting Mean Reported Proportion of Interviewers Asking about the Scholarly Project

	Proportion of Interviewers Asking about SP (%, SD)	***p***-value
**Type of Specialty**		0.2221
Primary Care	33% (28.1)	
Non-Primary Care	23% (25.6)	
**Competitiveness of Specialty**		0.2856
More Competitive	18% (26.3)	
Less Competitive	29% (29.9)	
**Publication Status**		0.9005
Published	27% (27.0)	
Not Published	26% (27.1)	
**Presentation Status**		0.1876
Presented	34% (30.4)	
Not Presented	23% (24.1)	
**Topic Relatedness to Specialty**		0.4553
Topic Related	30% (27.2)	
Topic Unrelated	24% (26.7)	
**Amount of Research Undertaken**		**0.0237**
Additional Research	50% (26.2)	
Only Scholarly Project	29% (28.4)	
**Type of Residency Programs**		**0.0054**
Almost All - More than Half Academic Programs	31% (27.9)	
None - Less than half Academic Programs	22% (25.5)	

Overall, 67% of students strongly agreed or agreed that it was helpful to have a scholarly project requirement as a component of their medical school curriculum. 19% of students neither agreed nor disagreed with this statement and only 14% stated that they disagreed.

## Discussion

This study provides unique insight into the impact of research experiences on the residency interview by showing that on average 40% of interviewers asked students about their research experiences. This supports the previously demonstrated observation in the literature that students with a greater number of research experiences are more likely to be successful in the NRMP match [7–10]. These findings also challenge applicants to view these experiences as more than simply a bullet on their resume but rather as a dynamic piece of their application about which they should anticipate questions during the interview process. Although this study suggests that interviewers inquire about research experiences less than half of the time, this knowledge provides value to students and advisors as they seek to better understand the role of research on the residency application. Research experiences are discussed during the residency interview at a similar frequency compared with volunteer experiences and work experiences.

All medical students at UACOMP are required to complete a longitudinal SP. Among the medical students surveyed in this study, approximately one-quarter (27%) of their interviewers utilized this as a discussion topic during their residency interview. This finding may encourage medical schools to consider adding programmatic objectives focused on student communication about their scholarly research. This may create opportunities for teaching students not only how to be investigators, but how to communicate their scholarly research in a compelling way. Additionally, a student’s chosen specialty, competitiveness of specialty and relatedness of SP topic to their chosen specialty had no significant impact on the proportion of interviewers inquiring about their SP. This may indicate that interviewers are more interested in the types of skills and traits acquired through scholarly research than the actual topic of the research. Furthermore, this observation may serve to diminish the notion that research is not important for students applying to primary care specialties. Overall, a mandatory scholarly project during medical school could provide a topic of conversation during the interview process regardless of the specialty students are applying for and the relatedness of their research topic to this specialty.

The literature suggests that students with a greater number of publications and presentations are more likely to be successful in the match than their peers [7–10]. This may lead students to believe that research is only valuable on their application if these milestones are obtained, however, the findings of this study suggest that research may be important as a discussion topic during the interview regardless of whether it received publication and presentation status. It is likely that interviewers see value in discussing these academic endeavors with students regardless of the project’s result. The impressive frequency of publication (48 and 78%) and presentation (33 and 48%) of students’ SP and additional non-SP research, respectively, should not be overlooked. Despite publication and presentation status not significantly impacting the number of interview questions a student received about research, the incorporation of a formal SP curriculum does appear to lead to increased achievement of these milestones. Although measuring the impact of this is beyond the scope of this study, this observation is in line with prior conclusions drawn by the University of Pittsburg [11].

There are two circumstances in which students may anticipate a greater number of interview questions about their SP. First, if the residency setting is academic the percentage of interviewers asking about a student’s SP increases from 33 to 50%. It is understandable that interviewers at academic programs may use research endeavors to learn more about an applicant’s attributes while interviewers at community programs may utilize alternative discussion topics to get to know the applicant. Second, students who undertake additional research beyond their mandated SP receive a greater number of interview questions about the topic compared to those students who do not undertake additional research, 50% compared to 29%, respectively. It is reasonable that with research experiences making up a more substantial piece of these student’s extracurricular activities, the SP, as a piece of the research portfolio, becomes a more frequent part of the interview discussion than students who only completed mandatory research requirements.

### Limitations

Although multiple interesting observations were revealed in our study, there are several limitations including a small sample size of residency applicants from only a single medical school. The retrospective nature of the survey lends itself to recall bias in which survey participants may not have been accurate in their estimations of the number of interviewers asking them about their experiences. There was range of one to 4 months between student completion of interviews and participation in the study survey. Furthermore, the study population is one-sided in that it only examines the experiences of the interviewee regarding discussions about research during the residency interview and does not evaluate the experience or intentions of the interviewer. Despite these limitations, this study provides unique quantitative observations about the topics of discussion during the residency interview. The findings of this study could help to guide future medical students within our institution regarding the impact of their scholarly project in the residency interview, and it is our hope that these findings may be useful for research programs at other schools of medicine as well.

## Conclusion

Student research experiences may serve as a frequent discussion topic during the residency interview. Approximately one-quarter of interviewers (27%) ask about the scholarly project regardless of type of specialty, competitiveness of specialty, relatedness of project topic to specialty, and publication/presentation status of project. Students with additional research experiences beyond their scholarly project may experience up to half of interviewers asking about their scholarly project whereas students applying to community programs with less academic/research focus may experience fewer questions about research compared to their peers applying to more academic programs. These findings have implications for medical students as they choose research projects and medical school administrators as they consider the impact of research on the success of their students in the residency match.

## Data Availability

The datasets used and/or analyzed during the current study are available from the corresponding author on reasonable request.

## Abbreviations

NRMP: National Residency Match Program
SP: Scholarly project
UACOMP: University of Arizona College of Medicine – Phoenix
SD: Standard deviation
MSPE: Medical Student Performance Evaluation
